# Neocolonialism and science diplomacy: lessons learned from the field and the way forward

**DOI:** 10.3389/fpubh.2024.1472421

**Published:** 2024-11-12

**Authors:** Rana Dajani, Rasha Bayoumi, Heather Flowe, Sarah Rockowitz, Laura Stevens, Abdullah Awad

**Affiliations:** ^1^Department of Biology and Biotechnology, Faculty of Science, The Hashemite University, Zarqa, Jordan; ^2^School of Psychology, University of Birmingham, Dubai, United Arab Emirates; ^3^School of Psychology, University of Birmingham, Edgbaston, United Kingdom; ^4^Institute for Critical Thought, Amman, Jordan

**Keywords:** science diplomacy, global south, neocolonialism, people of color, developing nations, decolonizing research

## Abstract

To level the playing field in the production of global knowledge, we need to understand the practical implications of colonial heritage and how it has disproportionately affected scientific discourse and the generation and utilization of scientific knowledge from and about the Global South. This article explores how research practitioners can level the playing field. We must think about how we can collectively change the narrative so that every emerging scientist from the Global South can flourish and have an equal opportunity to conduct research that is meaningful to them and their societies. We argue the time has come for innovative and flexible models allowing science diplomacy to integrate features of reflexive and inclusive governance in its very core structures.

## Introduction

According to the UK Royal Society and the American Association for the Advancement of Science document on science diplomacy that was published in 2010 (1) p. 19–22, science diplomacy can be defined to include three components:

- Diplomacy for science – the use of diplomatic action to facilitate international scientific collaboration, e.g., by negotiating R&D agreements and exchange programmes or enabling the establishment of international research infrastructures;
- Science for diplomacy – the use of science as a soft power to advance diplomatic objectives, e.g., for building bridges between nations and creating good will on which diplomatic relations can be built;
- Science in diplomacy – the direct support of diplomatic processes through science, e.g., by providing scientific advice and evidence to inform and support decision-making in foreign and security policies.

In other words Science diplomacy is a collection of practices encouraging the intersection of science and international relations, that has been of increasing relevance over the last few decades (2, 3). Currently the European Commission, UK Royal Society and AAAS are exploring updating the 2010 document (4).

The frameworks of science diplomacy within the context of colonial heritage need to be revised. This is particularly the case in the field of public health. While the interpretive social sciences have developed theoretical interventions and methodologies for gauging the shortcomings of sciences diplomacy in the Global South, there is a wide gap between decolonial discourse and action. In the series of reflections below, collated from researchers in public health, we argue that a more robust framework for science diplomacy must be developed and implemented at a large scale. Such a framework should prioritize the lived experience and modes of knowledge which exist in the underrepresented regions of the Global South, especially as they manifest with regards to public health.

If the COVID-19 pandemic has taught us anything about science diplomacy, it is that the discourse has left much to be desired when it comes to translating policy into action and the disproportionality with which this has been felt in the Global South. The pandemic highlights what had been known for decades: the translation and realization of science diplomacy is still plagued with remnants of neocolonialism (5). For instance, this was illustrated by Eurocentric and high-income country-specific response recommendations in combating the spread of the virus as suggested by the World Health Organization (WHO) (6). This included indiscriminate lockdown and social distancing procedures that did not account for cultural norms, country-specific population densities, and living conditions that could affect the implementation of these practices in the Global South.

## Neocolonialism throughout history

As researchers from the Global South and Global North, we have also witnessed and been subjected to numerous instances where the impact of neocolonialism is palpable. If we aim to level the playing field in the production of global knowledge, we need to understand the practical implications of colonial heritage and how it has affected the discourse, generation, and utilization of scientific knowledge from the Global South.

The complex research landscape in the Global South is further complicated by colonial heritage, and scientific diplomacy frameworks need to incorporate colonial history and its past impact and lingering effects. For example, interjecting equality in Global South research will require creative ways to ensure extractive practices are no longer the norm and new practices need to be incorporated to ensure we no longer have asymmetrical research relationships between countries and regions. Although overt extractive processes such as populating European museums with African and Asian artifacts might not be as frequent, implicit examples remain. While conducting fieldwork in Africa, one of the authors personally experienced neocolonial attitudes held by Western collaborators and the distrust felt by local collaborators and participants. The fear of ‘our’ stories and ‘our’ data being extracted to tell a story ‘about us’ is palpable, and one that needs to be addressed practically if we want to achieve equity in science diplomacy. One way in which the ‘telling of the story’ becomes more authentic is using local voices. We need to tell our stories through our researchers. While existing knowledge might help us by providing us with the necessary tools and methods to extract these stories, the days of Western extraction must come to an end.

Witnessing Western scientists reporting on their study of migrant populations and telling their story from the Western perspective today is painful, and adds salt to the postcolonial wound. There is clear re-traumatization of marginalized populations from the implicit judgment of behaviors as “inferior” as concluded through the lens of the Western scientist anthropologist ‘who knows best’ and is a ‘methodological expert’ due to their reputation as the world renowned ‘objective’ voice of science (7). A white European obstetrician, working in a Global North country, may tell the story of ‘ethnic’ immigrants, interpreting their cultural practices about menstruation from her lens. She described the immigrants’ discussion of their period as ‘dirty’ and a cause of isolation from family—her interpretation. However, the young researcher noted that in their culture, menstruation is considered a time to stay away from intercourse. The global expert, interpreting the information through her own lens, judged the participants as inferior. She recounts that narrative with her judgment intertwined so skillfully such that she is perceived by her peers to be the culturally sensitive scientist crusader aiming to enhance women’s lives globally. However, she is blissfully unaware of the young ‘ethnic’ immigrant scientist sitting in the audience whose face is at once transformed from admiring a role model to a fearful child being judged harshly by a parent as falling short of expectations. As a fellow researcher from the Global South, one of the authors who was in the room was able to support the young researcher through a science diplomacy approach by connecting her with international peers, amplifying her voice in global discussions, and showing her how her work could drive real policy change and make an impact beyond borders. This practical example highlights the need for us as research practitioners and scientists to reflect on how we can level the playing field. We need to think about how we can collectively change the narrative so every emerging scientist from the Global South can flourish and have an equal opportunity to conduct research that is meaningful to them and their societies.

## Neocolonialism in the present day

While many might believe that we are well on our way to achieving equity in accessing knowledge and science and technology resources, there remains an action discrepancy in science diplomacy, where policies might have changed, but they remain unactioned. As aforementioned, the COVID-19 pandemic has demonstrated this huge discrepancy between seemingly equitable policies and action on the ground related to response recommendations and the testing, distribution, and uptake of vaccines globally (6). More than ever, the lag in science and technology was laid bare and the consequences could not be ignored. As the body count increased, the inequitable access and affordability of vaccines and treatment disproportionately impacting the Global South became an evident example that we as global citizens had failed to deliver basic human rights, to deliver on the Sustainable Development Goals (SDGs) and to act on science diplomacy. Another stumbling block to science diplomacy has been the implicit idea that to level the playing field, the ‘West’ is still falling into an old colonial pastoral role that undermines local knowledge and expertise. This top-down approach can be viewed as patronizing and becomes an additional layer of distrust as it can be perceived as a postcolonial mechanism to exert soft power and insert Western ideas, propaganda, and interests. These fears are not unreasonable given postcolonial intergenerational trauma reverberates globally, and its impact has yet to be felt fully. Most recently, the war crimes and crimes against humanity perpetuated by Israel in Gaza have led South Africa to charge Israel with genocide at the International Court of Justice. Those crimes are militarily and diplomatically supported by Western governments, including the US, UK, Germany, and France, and they have shown how settler-colonial violence is still embedded in various levels of Western society, including among scientific communities (8, 9). As such, the voice of Palestinian scientists and those working on their behalf has been silenced and Western academic journals have regularly attempted to censure both the experience of Palestinians and the framing of their cause according to international humanitarian laws (10).

Many questions come to mind when exploring the possibility of using the soft power of science diplomacy in the context of armed conflict. For example, does scientific collaboration have the ability to influence state relations or do state relations determine the nature of scientific collaboration? What tools are available for scientists to engage these dynamics? We raise these questions in order to think the relationship between science diplomacy and politics, as well as to engage with new frameworks and models.

One important approach that comes to mind in this context is the Boycott, Divestment and Sanctions movement (11) The actual act of sanctioning is standard procedure by the UN in general and by the scientific community in particular to ensure accountability, a famous example of which is the boycott of the South African apartheid regime. This becomes relevant in the case of Israel, where the relationship between science and military technology is essential for the occupation (14–16). It is important to consider, moreover, that reshaping science diplomacy will have to take into account emerging multilateral power dynamics in the twenty-first century (17).

In this regard, one must also be aware that the rhetoric of Global North vs. Global South does not always hold. India is a case in point; the nation India is an important part of the Global South and has experienced the injurious effects of the British empire and yet it supports Israel militarily. Therefore the need for more nuanced analysis is an important point to keep in mind when speaking about decolonisation. Ambimbola and Pai write in an article about the decolonisation of global health: “To decolonise global health is to remove all forms of supremacy within all spaces of global health practice, within countries, between countries, and at the global level.” (16) p. 1627–1628.

By the same token, it is important to reflect on the limitations of well-meaning but politically complacent actors in the Global North. Moderate political stances on issues of science diplomacy may only maintain the status quo, leaving little space for honest conversations around structural and political issues. As Martin Luther King said in the context of the United States:

“First, I must confess that over the last few years I have been gravely disappointed with the white moderate. I have almost reached the regrettable conclusion that the Negro’s great stumbling block in the stride toward freedom is not the White Citizen’s Council-er or the Ku Klux Klanner, but the white moderate who is more devoted to “order” than to justice; who prefers a negative peace which is the absence of tension to a positive peace which is the presence of justice; who constantly says “I agree with you in the goal you seek, but I cannot agree with your methods of direct action;” who paternalistically feels he can set the timetable for another man’s freedom; who lives by the myth of time and who constantly advises the Negro to wait until a “more convenient season.” Shallow understanding from people of goodwill is more frustrating than absolute misunderstanding from people of ill will. Lukewarm acceptance is much more bewildering than outright rejection” (17).

Those interested in truly changing the narrative and enhancing access to knowledge, science and technology resources through science diplomacy should proceed with caution, and efforts to build bridges through science diplomacy should be grassroots and co-created with the relevant stakeholders in the Global South.

## The way forward

What lessons can we learn from the field? Success stories are evident from practice and literature, and we may learn from them, developing models and protocols for success. Action plans must be based on a true understanding of the mechanisms of change, of the translation of policy into truly equitable actions on the ground and felt by those most disadvantaged. One way to safeguard against the pitfalls mentioned and to ensure equitable scientific discourse and research practices is through the incorporation of local knowledge and expertise (20).

Some good practices in relation to the problem of extraction of data are recent advances in methodology to achieve a shared and participatory epistemic approach such as Fuzzy cognitive mapping, Bayesian belief networks, Participatory Systems Mapping, Rich pictures, System Dynamics: Theory of Change maps and other methods (19). We have employed Fuzzy cognitive mapping in understanding what empowerment means among Syrian refugee women in Jordan (20). In this study we were able to demonstrate a participatory approach to localizing knowledge and program evaluation through visual maps and scenarios of change. We have suggested that through science diplomacy one can contribute to promoting such good practices. We have recommended that improving scientific enquiry and public policy must be based on interventions which center the people affected on the ground.

Another example is the intricate relationship dynamics between scientists and the communities they study. When building these relationships, scientists do not pay attention to subtle undertones of perception of the different parties. As a scientist studying another population the relationship is transactional: “I want something from you, and I give you something in return.” While for equality the relationship should be communal “I am part of your community and together we want to understand a phenomenon and create solutions to solve it.” A scientist can only assume this mindset when the scientist is from the people being studied (22). Many times, the shortage of local scientists is cited as an excuse for not working with researchers from regional communities. This is despite the fact that almost every country has a diaspora which can be sought to support in finding relevant scientists and local partners. One way of implementing this is to advocate for UN bodies and International NGOs to include diaspora in decision making, program design and implementation in their respective countries. This will shift the narrative to create new and inclusive frameworks of systems in Global South research and practice (23).

Another important way in which equity according to science diplomacy can be achieved is through enhanced alignment between key sectors and stakeholders including higher education institutions, foreign policy makers, local government bodies, international organizations, and industry partners in science, technology, and innovation. This alignment will ensure efforts are not fragmented or duplicated and more importantly, significant tasks do not fall between the cracks. Perhaps addressing and facilitating this coordinated approach could be the link between science diplomacy discourse and actionable outputs. In this way, diplomatic efforts can synergistically enable and augment the scientific collaborations addressing existing gaps. This will enable Global South to move from the place of being the target of, and audience, to scientific knowledge, to becoming an equal participating partner in its production and the sequelae that result from the enhanced contextually relevant knowledge. Two examples of this are explained in the next section.

Guidelines for conducting research and developing and applying therapies are based on ethical values. Ethical values combine cultural, religious, political, and social bases in their formation. Yet in many instances we observe a lack of inclusion of cultural and religious backgrounds. An example is stem cell guidelines governing research and therapy. Ethical bodies such as the ISSCR do not have scientists of the Muslim faith (24). This is important not only to advance science, especially in the case of faith-based values, but also to respect the communities who are affected by the ethical guidelines such as patients, families, practitioners, and scientists. The inclusion of scientists from these communities is necessary to legitimately achieve science diplomacy as a part of it is inclusivity at the level of policy making and guideline forming for governing science ethics.

Enhanced coordination between stakeholders and across sectors are promising approaches to increasing equity according to science diplomacy, however these partnerships must be carefully planned and managed for real progress to be made. Inequity in these partnerships is certainly prevalent as has been found by a study on a collaborative research project that had Africa-based researchers expressing concerns over power asymmetries when collaborating with Global North researchers. They stated that these studies placed them as data collectors for North research rather than legitimate collaborators (25). This demonstrates the current lack of equity in the Global South. What we have listed are some examples of how to address epistemic injustice and achieve shared knowledge production; to include diaspora researchers and all faiths in decision making; and to enhance alignment between key sectors and stakeholders. In addition to these factors we should also consider interventions that challenge the power structure itself. However, there are risks to these approaches that are worth mentioning. One must be careful that the inclusion of representatives of the diaspora does not end up in tokenism.

Another issue we raise is when LMIC partners are involved, extra care must be taken because reliance on international funding may influence an LMIC researcher’s life more than their partner in a higher-income country. One such example is the Global Challenges Research fund.

Despite noble intentions at the start, cuts to the Global Challenges Research Fund (GCRF) once again reflect the struggles international scholars face when trying to work collaboratively to reduce global inequality working toward the United Nations’ Sustainable Development Goals (26). A main tenet of the GCRF is to focus its work on the ‘developing world,’ yet the sudden reduction in aid means strong partnerships which had been built thanks to this fund, and the international partners in LMICs that had come to rely on this funding for their work, were stripped back and forced to operate with a steep reduction in resources.

Initiatives such as those in Jordan and Palestine have come to rely on GCRF funding. These protect refugees from the intergenerational impacts of forced displacement and trauma. Some are in Kenya led by survivors of sexual violence to investigate the cumulative harms of people struggling to access post-sexual violence medical care. Additionally, work in Rwanda and Lebanon studying the histories of violence and discrimination are hugely reliant on international funding, both to keep their projects running and to enhance access to knowledge for on-the-ground scholars. These cuts could mean the premature end of some of these projects, or at least that international partners who had come to depend on this funding no longer have the resources to continue forward as planned.

With these effects in mind, the GCRF cannot rightly say that it is working to reduce global inequality and that the cutting of this fund became a side effect of pandemic politics when their funds could be used to research and prevent future pandemics. This indicates that once again, despite promises to focus on working in developing countries, the UK government has prioritized themselves over everyone else, failing to even consider the UK-based early career researchers who may depend on this funding for their livelihoods. Adding insult to harm is that the UK government is currently supporting Israel with military arms to commit war crimes which includes targeting facilities funded by the UK government and GCRF like the Palestine Trauma Center in Gaza, while simultaneously talking about funding research in development of these regions (27, 28). This exposure to violence and the internal displacement of more than 1.9 million people within the region coupled with the destruction of hospitals, water facilities and public toilets has led to prevalence of water-borne diseases, dehydration, and mental health issues within the Gazan population (29). The double standard is expected given the state of the world as we have described earlier yet must be called out if we are to move toward a more just and equal world.

As previously discussed, there have seen several models proposed to help translate science diplomacy. We suggest that the time has come for us to collectively consider the effective mechanism of those models and develop a unified framework addressing science diplomacy action discrepancy (30).

## Discussion

This article has reflected on science diplomacy and its development in the context of entrenched global inequalities and colonial legacies. It has highlighted some concrete examples of how large-scale projects set on bridging knowledge gaps between the Global North and the Global South end up reproducing patterns of subjugation instead of co-ownership in knowledge production.

Lofty rhetoric may emphasize co-development of research project designs and values. Yet, decision-making in funding priorities and cuts remains largely monopolized by institutions and governments of the Global North, which rely on their own foreign policy prerogatives. In that regard, the promise to ‘partner’ with stakeholders in the Global South as active research agents and protagonists remains unfulfilled and flimsy. Considering financial cuts and downsized research budgets, researchers in LMICs relying on funds from the Global North have become vulnerable and unable to implement the very goals that they have signed up for at the outset.

Developing synergistic projects that transcend the Global North/Global South binary does not merely consist of having researchers from LMICs on board. Rather, a profound rethinking of ethical values driving knowledge exchange and inclusion is at stake. How can we enhance equity and inclusion in science diplomacy? How can governments’ financial power in research be turned into a force for good, rather than a tool of ‘neocolonialism?’ How can researchers be positioned as *protagonists* rather than passive *recipients* of funds irrespective of geographies, geo-economic fluctuations, and state hierarchies in the international system? These are some of the looming challenges.

Concurrent crises from the pandemic to Russia’s invasion of Ukraine and the West’s support of the unfolding genocide in Gaza, as well as their repercussions on funding priorities and research, reveal that science diplomacy is a particularly marginalized policy field. In the context of these concomitant crises, we call for increasing multilateral, interdisciplinary, and transversal partnerships that seek to rethink science diplomacy from the grassroots. We also call for research that utilizes innovative models allowing science diplomacy to integrate features of reflexive and inclusive governance in its very core structures. One starting point would be to learn and derive broader insights from small-scale research projects that have included local communities as research stakeholders rather than targets in all research stages from inception to implementation with the goal of preserving equality and dignity for all.

## Data Availability

The original contributions presented in the study are included in the article/supplementary material, further inquiries can be directed to the corresponding author.
